# Supplementary material for: Electron probe microanalysis of the elemental composition of phytoliths from woody bamboo species
Source: PLoS One. 2022 Jul 5;17(7):e0270842. doi: 10.1371/journal.pone.0270842 (PMC9255755; doi:10.1371/journal.pone.0270842)
Supplement: S1 Table — A. EPMA (using NCA and Fix-C method) results (elemental compositions and total values (wt.%)) of different phytolith morphotypes in leaves of bamboo species from three genera. B. Average elemental compositions and total values (wt.%) (using Fix-C and NCA method) of phytoliths in leaves of bamboo species from three genera. C. The elemental content of dominant phytolith morphologies from Dendrocalamus ronganensis leaves determined by EPMA with three methods. (DOCX) [file pone.0270842.s001.docx]

**S1 Table. EPMA results.**

**A.** EPMA (using NCA and Fix-C method) results (elemental compositions and total values (wt.%)) of different phytolith morphotypes in leaves of bamboo species from three genera.

| Genus | Species | Method | Phytolith morphology | Phytolith particles | EPMA results | | | | | | | | | | | |
| --- | --- | --- | --- | --- | --- | --- | --- | --- | --- | --- | --- | --- | --- | --- | --- | --- |
|  |  |  |  |  |  |  |  |  |  |  |  |  |  |  |  |  |
|  |  |  |  |  | SiO2 | Na2O | Al2O3 | MgO | CaO | MnO | Cr2O3 | P2O5 | K2O | FeO | C | Total |
|  |  |  |  |  |  |  |  |  |  |  |  |  |  |  |  |  |
| *Dendrocalamus* | Dendrocalamus brandisii (Munro) Kurz | NCA | Grass silica short cell | 10 | 83.97 | 0.02 | 0.05 | 0.02 | 0.03 | 0.06 | 0.02 | 0.01 | 0.10 | 0.01 | 0.00 | 84.30 |
|  |  |  | ELONGATE | 7 | 88.32 | 0.03 | 0.05 | 0.03 | 0.13 | 0.38 | 0.01 | 0.01 | 0.10 | 0.00 | 0.00 | 89.07 |
|  |  |  | BULLIFORM FLABELLATE | 7 | 84.58 | 0.02 | 0.04 | 0.02 | 0.02 | 0.06 | 0.03 | 0.02 | 0.11 | 0.01 | 0.00 | 84.90 |
|  |  |  |  | 24 | 85.42 | 0.02 | 0.05 | 0.02 | 0.06 | 0.16 | 0.02 | 0.01 | 0.10 | 0.01 | 0.00 | 85.87 |
|  |  | Fix-C | Grass silica short cell | 10 | 82.86 | 0.02 | 0.08 | 0.02 | 0.04 | 0.08 | 0.04 | 0.01 | 0.11 | 0.01 | 2.70 | 85.96 |
|  |  |  | ELONGATE | 7 | 88.10 | 0.04 | 0.05 | 0.03 | 0.14 | 0.36 | 0.02 | 0.02 | 0.09 | 0.01 | 2.70 | 91.55 |
|  |  |  | BULLIFORM FLABELLATE | 7 | 86.41 | 0.03 | 0.04 | 0.02 | 0.04 | 0.07 | 0.02 | 0.01 | 0.12 | 0.02 | 2.70 | 89.47 |
|  |  |  |  | 24 | 85.42 | 0.03 | 0.06 | 0.02 | 0.07 | 0.16 | 0.02 | 0.01 | 0.10 | 0.01 | 2.70 | 88.61 |
|  | Dendrocalamus farinosus (Keng et Keng. f.) Chia et H. L. Fung | NCA | Grass silica short cell | 10 | 85.30 | 0.02 | 0.13 | 0.02 | 0.09 | 0.04 | 0.03 | 0.01 | 0.13 | 0.00 | 0.00 | 85.76 |
|  |  |  | ELONGATE | 7 | 84.49 | 0.08 | 0.82 | 0.02 | 0.11 | 0.01 | 0.03 | 0.01 | 0.22 | 0.01 | 0.00 | 85.78 |
|  |  |  | BULLIFORM FLABELLATE | 7 | 85.85 | 0.03 | 0.21 | 0.03 | 0.13 | 0.01 | 0.05 | 0.01 | 0.15 | 0.01 | 0.00 | 86.47 |
|  |  |  |  | 24 | 85.22 | 0.04 | 0.35 | 0.02 | 0.10 | 0.02 | 0.03 | 0.01 | 0.16 | 0.01 | 0.00 | 85.97 |
|  |  | Fix-C | Grass silica short cell | 10 | 80.77 | 0.02 | 0.85 | 0.02 | 0.09 | 0.04 | 0.01 | 0.01 | 0.09 | 0.01 | 1.40 | 83.32 |
|  |  |  | ELONGATE | 7 | 89.54 | 0.05 | 0.17 | 0.02 | 0.08 | 0.01 | 0.02 | 0.01 | 0.13 | 0.00 | 1.40 | 91.43 |
|  |  |  | BULLIFORM FLABELLATE | 7 | 83.92 | 0.13 | 0.18 | 0.02 | 0.14 | 0.01 | 0.03 | 0.01 | 0.15 | 0.00 | 1.40 | 85.98 |
|  |  |  |  | 24 | 84.25 | 0.06 | 0.46 | 0.02 | 0.10 | 0.02 | 0.02 | 0.01 | 0.12 | 0.01 | 1.40 | 86.46 |
|  | Dendrocalamus minor (McClure) Chia et H. L. Fung | NCA | Grass silica short cell | 10 | 85.43 | 0.02 | 0.08 | 0.01 | 0.03 | 0.02 | 0.03 | 0.00 | 0.17 | 0.01 | 0.00 | 85.81 |
|  |  |  | ELONGATE | 7 | 79.31 | 0.01 | 0.08 | 0.08 | 0.41 | 0.06 | 0.02 | 0.01 | 0.07 | 0.02 | 0.00 | 80.06 |
|  |  |  | BULLIFORM FLABELLATE | 7 | 84.15 | 0.02 | 0.05 | 0.02 | 0.22 | 0.02 | 0.02 | 0.01 | 0.26 | 0.00 | 0.00 | 84.74 |
|  |  |  |  | 24 | 83.27 | 0.02 | 0.07 | 0.03 | 0.19 | 0.03 | 0.02 | 0.01 | 0.17 | 0.01 | 0.00 | 83.82 |
|  |  | Fix-C | Grass silica short cell | 10 | 86.95 | 0.02 | 0.25 | 0.01 | 0.04 | 0.05 | 0.04 | 0.01 | 0.17 | 0.02 | 1.40 | 88.97 |
|  |  |  | ELONGATE | 7 | 83.01 | 0.01 | 0.06 | 0.09 | 0.41 | 0.08 | 0.02 | 0.00 | 0.07 | 0.01 | 1.40 | 85.16 |
|  |  |  | BULLIFORM FLABELLATE | 7 | 83.08 | 0.02 | 0.04 | 0.01 | 0.21 | 0.00 | 0.02 | 0.01 | 0.28 | 0.01 | 1.40 | 85.09 |
|  |  |  |  | 24 | 84.67 | 0.02 | 0.13 | 0.04 | 0.20 | 0.04 | 0.03 | 0.01 | 0.17 | 0.01 | 1.40 | 86.72 |
|  | Dendrocalamus giganteus Munro | NCA | Grass silica short cell | 10 | 86.76 | 0.07 | 0.07 | 0.04 | 0.08 | 0.07 | 0.03 | 0.01 | 0.28 | 0.00 | 0.00 | 87.40 |
|  |  |  | ELONGATE | 7 | 85.80 | 0.07 | 0.06 | 0.01 | 0.17 | 0.02 | 0.04 | 0.01 | 0.23 | 0.00 | 0.00 | 86.42 |
|  |  |  | BULLIFORM FLABELLATE | 7 | 85.75 | 0.05 | 0.03 | 0.05 | 0.15 | 0.03 | 0.02 | 0.01 | 0.18 | 0.01 | 0.00 | 86.28 |
|  |  |  |  | 24 | 86.18 | 0.06 | 0.05 | 0.03 | 0.13 | 0.05 | 0.03 | 0.01 | 0.24 | 0.00 | 0.00 | 86.79 |
|  |  | Fix-C | Grass silica short cell | 10 | 86.72 | 0.06 | 0.06 | 0.03 | 0.07 | 0.07 | 0.03 | 0.01 | 0.22 | 0.01 | 2.40 | 89.68 |
|  |  |  | ELONGATE | 7 | 86.74 | 0.05 | 0.03 | 0.02 | 0.20 | 0.02 | 0.05 | 0.00 | 0.21 | 0.01 | 2.40 | 89.72 |
|  |  |  | BULLIFORM FLABELLATE | 7 | 86.82 | 0.03 | 0.02 | 0.03 | 0.16 | 0.03 | 0.01 | 0.01 | 0.15 | 0.00 | 2.40 | 89.68 |
|  |  |  |  | 24 | 86.75 | 0.05 | 0.04 | 0.03 | 0.13 | 0.04 | 0.03 | 0.01 | 0.20 | 0.01 | 2.40 | 89.69 |
|  | Dendrocalamus pachystachys Hsueh et D. Z. Li | NCA | Grass silica short cell | 10 | 81.10 | 0.02 | 0.06 | 0.03 | 0.08 | 0.16 | 0.01 | 0.01 | 0.13 | 0.01 | 0.00 | 81.60 |
|  |  |  | ELONGATE | 7 | 85.29 | 0.02 | 0.03 | 0.03 | 0.14 | 0.18 | 0.01 | 0.00 | 0.17 | 0.00 | 0.00 | 85.88 |
|  |  |  | BULLIFORM FLABELLATE | 8 | 84.02 | 0.02 | 0.03 | 0.03 | 0.20 | 0.06 | 0.02 | 0.00 | 0.13 | 0.01 | 0.00 | 84.52 |
|  |  |  |  | 25 | 83.21 | 0.02 | 0.04 | 0.03 | 0.13 | 0.13 | 0.01 | 0.00 | 0.14 | 0.01 | 0.00 | 83.73 |
|  |  | Fix-C | Grass silica short cell | 10 | 80.73 | 0.02 | 0.04 | 0.03 | 0.07 | 0.12 | 0.03 | 0.01 | 0.12 | 0.01 | 1.70 | 82.87 |
|  |  |  | ELONGATE | 7 | 84.56 | 0.02 | 0.04 | 0.03 | 0.13 | 0.15 | 0.03 | 0.01 | 0.17 | 0.00 | 1.70 | 86.85 |
|  |  |  | BULLIFORM FLABELLATE | 8 | 84.33 | 0.02 | 0.04 | 0.03 | 0.20 | 0.05 | 0.01 | 0.01 | 0.15 | 0.00 | 1.70 | 86.54 |
|  |  |  |  | 25 | 82.96 | 0.02 | 0.04 | 0.03 | 0.13 | 0.11 | 0.02 | 0.01 | 0.14 | 0.01 | 1.70 | 85.16 |
|  | Dendrocalamopsis vario-striata (W. T. Lin)Keng f. | NCA | Grass silica short cell | 10 | 87.44 | 0.03 | 0.07 | 0.02 | 0.03 | 0.03 | 0.04 | 0.01 | 0.22 | 0.01 | 0.00 | 87.89 |
|  |  |  |  |  |  |  |  |  |  |  |  |  |  |  |  |  |
|  |  |  | ELONGATE | 7 | 84.28 | 0.03 | 0.04 | 0.04 | 0.11 | 0.01 | 0.02 | 0.01 | 0.14 | 0.01 | 0.00 | 84.71 |
|  |  |  | BULLIFORM FLABELLATE | 7 | 83.68 | 0.06 | 0.03 | 0.08 | 0.17 | 0.01 | 0.01 | 0.01 | 0.25 | 0.02 | 0.00 | 84.31 |
|  |  |  |  | 24 | 85.42 | 0.04 | 0.05 | 0.04 | 0.10 | 0.02 | 0.03 | 0.01 | 0.21 | 0.01 | 0.00 | 85.92 |
|  |  | Fix-C | Grass silica short cell | 10 | 86.01 | 0.04 | 0.06 | 0.02 | 0.04 | 0.04 | 0.03 | 0.01 | 0.17 | 0.00 | 2.00 | 88.42 |
|  |  |  |  |  |  |  |  |  |  |  |  |  |  |  |  |  |
|  |  |  | ELONGATE | 7 | 83.34 | 0.05 | 0.05 | 0.04 | 0.12 | 0.01 | 0.01 | 0.01 | 0.16 | 0.01 | 2.00 | 85.79 |
|  |  |  | BULLIFORM FLABELLATE | 7 | 83.03 | 0.06 | 0.03 | 0.09 | 0.16 | 0.01 | 0.02 | 0.00 | 0.24 | 0.00 | 2.00 | 85.65 |
|  |  |  |  | 24 | 84.36 | 0.05 | 0.05 | 0.05 | 0.10 | 0.02 | 0.02 | 0.01 | 0.19 | 0.00 | 2.00 | 86.84 |
|  | Dendrocalamus sapidus Q. H. Dar et D. Y. Huang | NCA | Grass silica short cell | 12 | 85.39 | 0.03 | 0.06 | 0.01 | 0.02 | 0.04 | 0.01 | 0.01 | 0.15 | 0.01 | 0.00 | 85.74 |
|  |  |  |  |  |  |  |  |  |  |  |  |  |  |  |  |  |
|  |  |  | ELONGATE | 7 | 84.85 | 0.06 | 0.20 | 0.02 | 0.12 | 0.01 | 0.01 | 0.01 | 0.14 | 0.01 | 0.00 | 85.43 |
|  |  |  | BULLIFORM FLABELLATE | 7 | 82.61 | 0.02 | 0.03 | 0.05 | 0.18 | 0.08 | 0.03 | 0.00 | 0.09 | 0.01 | 0.00 | 83.09 |
|  |  |  |  | 26 | 84.50 | 0.04 | 0.09 | 0.02 | 0.09 | 0.04 | 0.02 | 0.00 | 0.13 | 0.01 | 0.00 | 84.94 |
|  |  | Fix-C | Grass silica short cell | 12 | 84.16 | 0.03 | 0.07 | 0.01 | 0.02 | 0.05 | 0.03 | 0.02 | 0.12 | 0.01 | 3.30 | 87.81 |
|  |  |  |  |  |  |  |  |  |  |  |  |  |  |  |  |  |
|  |  |  | ELONGATE | 7 | 84.35 | 0.05 | 0.14 | 0.02 | 0.09 | 0.02 | 0.03 | 0.01 | 0.15 | 0.02 | 3.30 | 88.17 |
|  |  |  | BULLIFORM FLABELLATE | 7 | 82.73 | 0.03 | 0.03 | 0.04 | 0.20 | 0.07 | 0.03 | 0.01 | 0.09 | 0.01 | 3.30 | 86.55 |
|  |  |  |  | 26 | 83.82 | 0.04 | 0.08 | 0.02 | 0.09 | 0.05 | 0.03 | 0.01 | 0.12 | 0.01 | 3.30 | 87.57 |
| *Bambusa* | Bambusa eutuldoides McClure | NCA | Grass silica short cell | 10 | 83.48 | 0.05 | 0.15 | 0.03 | 0.10 | 0.03 | 0.04 | 0.01 | 0.21 | 0.01 | 0.00 | 84.09 |
|  |  |  | ELONGATE | 7 | 84.49 | 0.06 | 0.44 | 0.04 | 0.19 | 0.13 | 0.12 | 0.01 | 0.23 | 0.01 | 0.00 | 85.71 |
|  |  |  | BULLIFORM FLABELLATE | 7 | 88.23 | 0.07 | 0.07 | 0.06 | 0.16 | 0.14 | 0.02 | 0.01 | 0.32 | 0.01 | 0.00 | 89.07 |
|  |  |  |  | 24 | 85.16 | 0.06 | 0.21 | 0.04 | 0.14 | 0.09 | 0.05 | 0.01 | 0.25 | 0.01 | 0.00 | 86.02 |
|  |  | Fix-C | Grass silica short cell | 10 | 82.14 | 0.04 | 0.14 | 0.02 | 0.11 | 0.02 | 0.04 | 0.01 | 0.15 | 0.01 | 3.80 | 86.48 |
|  |  |  | ELONGATE | 7 | 85.57 | 0.08 | 0.36 | 0.05 | 0.23 | 0.18 | 0.11 | 0.01 | 0.25 | 0.00 | 3.80 | 90.64 |
|  |  |  | BULLIFORM FLABELLATE | 7 | 87.96 | 0.06 | 0.26 | 0.06 | 0.19 | 0.11 | 0.03 | 0.01 | 0.26 | 0.01 | 3.80 | 92.73 |
|  |  |  |  | 24 | 84.84 | 0.06 | 0.24 | 0.04 | 0.17 | 0.09 | 0.06 | 0.01 | 0.21 | 0.01 | 3.80 | 89.52 |
|  | Bambusa chungii McClure | NCA | Grass silica short cell | 10 | 84.26 | 0.04 | 0.13 | 0.06 | 0.05 | 0.02 | 0.02 | 0.01 | 0.23 | 0.01 | 0.00 | 84.83 |
|  |  |  |  |  |  |  |  |  |  |  |  |  |  |  |  |  |
|  |  |  | ELONGATE | 7 | 85.14 | 0.04 | 0.22 | 0.10 | 0.10 | 0.02 | 0.03 | 0.02 | 0.18 | 0.00 | 0.00 | 85.85 |
|  |  |  | BULLIFORM FLABELLATE | 7 | 85.14 | 0.03 | 0.04 | 0.13 | 0.10 | 0.01 | 0.01 | 0.01 | 0.11 | 0.02 | 0.00 | 85.60 |
|  |  |  |  | 24 | 84.78 | 0.04 | 0.13 | 0.09 | 0.08 | 0.02 | 0.02 | 0.01 | 0.18 | 0.01 | 0.00 | 85.35 |
|  |  | Fix-C | Grass silica short cell | 10 | 86.22 | 0.03 | 0.08 | 0.06 | 0.04 | 0.04 | 0.01 | 0.01 | 0.16 | 0.01 | 2.30 | 88.95 |
|  |  |  |  |  |  |  |  |  |  |  |  |  |  |  |  |  |
|  |  |  | ELONGATE | 7 | 86.21 | 0.03 | 0.11 | 0.11 | 0.10 | 0.01 | 0.02 | 0.01 | 0.14 | 0.01 | 2.30 | 89.05 |
|  |  |  | BULLIFORM FLABELLATE | 7 | 85.53 | 0.02 | 0.04 | 0.14 | 0.11 | 0.02 | 0.02 | 0.01 | 0.11 | 0.01 | 2.30 | 88.28 |
|  |  |  |  | 24 | 86.01 | 0.03 | 0.08 | 0.10 | 0.08 | 0.02 | 0.02 | 0.01 | 0.14 | 0.01 | 2.30 | 88.78 |
|  | Bambusa multiplex (Lour.) Raeusch. ex Schult. 'Fernleaf' R. A. Young | NCA | Grass silica short cell | 11 | 84.80 | 0.01 | 0.09 | 0.02 | 0.06 | 0.05 | 0.04 | 0.01 | 0.12 | 0.00 | 0.00 | 85.19 |
|  |  |  |  |  |  |  |  |  |  |  |  |  |  |  |  |  |
|  |  |  | ELONGATE | 7 | 83.15 | 0.03 | 0.23 | 0.01 | 0.12 | 0.01 | 0.02 | 0.01 | 0.22 | 0.01 | 0.00 | 83.81 |
|  |  |  | BULLIFORM FLABELLATE | 7 | 86.44 | 0.02 | 0.05 | 0.02 | 0.09 | 0.02 | 0.02 | 0.01 | 0.22 | 0.01 | 0.00 | 86.89 |
|  |  |  |  | 25 | 84.80 | 0.02 | 0.12 | 0.01 | 0.08 | 0.03 | 0.03 | 0.01 | 0.18 | 0.01 | 0.00 | 85.28 |
|  |  | Fix-C | Grass silica short cell | 11 | 86.26 | 0.01 | 0.05 | 0.02 | 0.06 | 0.05 | 0.01 | 0.01 | 0.11 | 0.02 | 3.00 | 89.59 |
|  |  |  |  |  |  |  |  |  |  |  |  |  |  |  |  |  |
|  |  |  | ELONGATE | 7 | 86.81 | 0.02 | 0.11 | 0.01 | 0.13 | 0.03 | 0.02 | 0.01 | 0.22 | 0.00 | 3.00 | 90.37 |
|  |  |  | BULLIFORM FLABELLATE | 7 | 84.49 | 0.01 | 0.04 | 0.01 | 0.10 | 0.01 | 0.03 | 0.02 | 0.20 | 0.00 | 3.00 | 87.91 |
|  |  |  |  | 25 | 85.92 | 0.02 | 0.06 | 0.01 | 0.09 | 0.03 | 0.02 | 0.02 | 0.16 | 0.01 | 3.00 | 89.34 |
|  | Bambusa textilis McClure | NCA | Grass silica short cell | 10 | 85.18 | 0.02 | 0.14 | 0.01 | 0.04 | 0.10 | 0.03 | 0.01 | 0.18 | 0.01 | 0.00 | 85.72 |
|  |  |  | ELONGATE | 7 | 87.63 | 0.02 | 0.07 | 0.03 | 0.11 | 0.03 | 0.02 | 0.01 | 0.13 | 0.00 | 0.00 | 88.07 |
|  |  |  | BULLIFORM FLABELLATE | 7 | 86.78 | 0.02 | 0.18 | 0.02 | 0.19 | 0.04 | 0.01 | 0.00 | 0.14 | 0.01 | 0.00 | 87.39 |
|  |  |  |  | 24 | 86.36 | 0.02 | 0.13 | 0.02 | 0.10 | 0.06 | 0.02 | 0.01 | 0.15 | 0.01 | 0.00 | 86.89 |
|  |  | Fix-C | Grass silica short cell | 10 | 85.35 | 0.01 | 0.23 | 0.02 | 0.04 | 0.10 | 0.03 | 0.01 | 0.13 | 0.01 | 2.50 | 88.43 |
|  |  |  | ELONGATE | 7 | 87.14 | 0.01 | 0.06 | 0.03 | 0.12 | 0.06 | 0.04 | 0.01 | 0.11 | 0.01 | 2.50 | 90.10 |
|  |  |  | BULLIFORM FLABELLATE | 7 | 85.97 | 0.03 | 0.63 | 0.02 | 0.21 | 0.05 | 0.01 | 0.01 | 0.10 | 0.01 | 2.50 | 89.53 |
|  |  |  |  | 24 | 86.05 | 0.02 | 0.30 | 0.02 | 0.11 | 0.07 | 0.03 | 0.01 | 0.11 | 0.01 | 2.50 | 89.24 |
|  | Bambusa remotiflora Kuntze | NCA | Grass silica short cell | 12 | 83.43 | 0.05 | 0.48 | 0.01 | 0.05 | 0.04 | 0.06 | 0.00 | 0.16 | 0.01 | 0.00 | 84.29 |
|  |  |  | ELONGATE | 7 | 82.63 | 0.02 | 0.06 | 0.01 | 0.16 | 0.11 | 0.10 | 0.01 | 0.11 | 0.01 | 0.00 | 83.22 |
|  |  |  | BULLIFORM FLABELLATE | 7 | 85.37 | 0.02 | 0.56 | 0.01 | 0.12 | 0.13 | 0.09 | 0.00 | 0.09 | 0.01 | 0.00 | 86.41 |
|  |  |  |  | 26 | 83.74 | 0.03 | 0.39 | 0.01 | 0.10 | 0.08 | 0.08 | 0.01 | 0.13 | 0.01 | 0.00 | 84.57 |
|  |  | Fix-C | Grass silica short cell | 12 | 84.67 | 0.02 | 0.17 | 0.01 | 0.05 | 0.04 | 0.05 | 0.01 | 0.08 | 0.01 | 1.80 | 86.89 |
|  |  |  | ELONGATE | 7 | 83.52 | 0.02 | 0.56 | 0.01 | 0.18 | 0.11 | 0.05 | 0.01 | 0.11 | 0.01 | 1.80 | 86.38 |
|  |  |  | BULLIFORM FLABELLATE | 7 | 90.35 | 0.02 | 0.12 | 0.01 | 0.11 | 0.12 | 0.07 | 0.01 | 0.13 | 0.00 | 1.80 | 92.73 |
|  |  |  |  | 26 | 85.89 | 0.02 | 0.26 | 0.01 | 0.10 | 0.08 | 0.05 | 0.01 | 0.10 | 0.01 | 1.80 | 88.33 |
|  | Bambusa blumeana J. A. et J. H. Schult. f | NCA | Grass silica short cell | 9 | 86.07 | 0.05 | 0.07 | 0.02 | 0.10 | 0.01 | 0.04 | 0.02 | 0.23 | 0.01 | 0.00 | 86.62 |
|  |  |  |  |  |  |  |  |  |  |  |  |  |  |  |  |  |
|  |  |  | ELONGATE | 8 | 87.35 | 0.08 | 0.08 | 0.01 | 0.06 | 0.01 | 0.03 | 0.01 | 0.24 | 0.00 | 0.00 | 87.86 |
|  |  |  | BULLIFORM FLABELLATE | 7 | 82.33 | 0.05 | 0.07 | 0.02 | 0.26 | 0.01 | 0.01 | 0.01 | 0.15 | 0.01 | 0.00 | 82.92 |
|  |  |  |  | 24 | 85.41 | 0.06 | 0.07 | 0.02 | 0.13 | 0.01 | 0.03 | 0.01 | 0.21 | 0.01 | 0.00 | 85.95 |
|  |  | Fix-C | Grass silica short cell | 9 | 85.03 | 0.05 | 0.03 | 0.01 | 0.07 | 0.01 | 0.05 | 0.02 | 0.19 | 0.01 | 3.00 | 88.48 |
|  |  |  |  |  |  |  |  |  |  |  |  |  |  |  |  |  |
|  |  |  | ELONGATE | 8 | 86.05 | 0.09 | 0.05 | 0.01 | 0.08 | 0.01 | 0.02 | 0.01 | 0.26 | 0.01 | 3.00 | 89.63 |
|  |  |  | BULLIFORM FLABELLATE | 7 | 85.56 | 0.04 | 0.02 | 0.02 | 0.25 | 0.02 | 0.02 | 0.02 | 0.14 | 0.01 | 3.00 | 89.09 |
|  |  |  |  | 24 | 85.53 | 0.06 | 0.04 | 0.01 | 0.12 | 0.01 | 0.03 | 0.02 | 0.20 | 0.01 | 3.00 | 89.04 |
|  | Bambusa contracta Chia et H. L. Fung | NCA | Grass silica short cell | 9 | 78.93 | 0.01 | 0.19 | 0.01 | 0.01 | 0.02 | 0.06 | 0.01 | 0.10 | 0.00 | 0.00 | 79.35 |
|  |  |  |  |  |  |  |  |  |  |  |  |  |  |  |  |  |
|  |  |  | ELONGATE | 7 | 85.99 | 0.04 | 0.13 | 0.02 | 0.11 | 0.05 | 0.04 | 0.02 | 0.25 | 0.01 | 0.00 | 86.66 |
|  |  |  | BULLIFORM FLABELLATE | 8 | 83.83 | 0.02 | 0.08 | 0.01 | 0.08 | 0.09 | 0.02 | 0.01 | 0.10 | 0.01 | 0.00 | 84.24 |
|  |  |  |  | 24 | 82.62 | 0.02 | 0.14 | 0.01 | 0.06 | 0.05 | 0.04 | 0.01 | 0.14 | 0.01 | 0.00 | 83.11 |
|  |  | Fix-C | Grass silica short cell | 9 | 82.61 | 0.02 | 0.16 | 0.01 | 0.02 | 0.04 | 0.05 | 0.01 | 0.14 | 0.01 | 1.80 | 84.84 |
|  |  |  |  |  |  |  |  |  |  |  |  |  |  |  |  |  |
|  |  |  | ELONGATE | 7 | 83.35 | 0.03 | 0.12 | 0.02 | 0.10 | 0.07 | 0.01 | 0.01 | 0.17 | 0.01 | 1.80 | 85.69 |
|  |  |  | BULLIFORM FLABELLATE | 8 | 85.59 | 0.02 | 0.15 | 0.01 | 0.07 | 0.10 | 0.02 | 0.00 | 0.12 | 0.01 | 1.80 | 87.89 |
|  |  |  |  | 24 | 83.82 | 0.02 | 0.14 | 0.01 | 0.06 | 0.07 | 0.03 | 0.01 | 0.14 | 0.01 | 1.80 | 86.10 |
|  | Bambusa albo-lineata Chia | NCA | Grass silica short cell | 10 | 85.03 | 0.02 | 0.09 | 0.02 | 0.04 | 0.03 | 0.04 | 0.01 | 0.16 | 0.00 | 0.00 | 85.45 |
|  |  |  | ELONGATE | 7 | 85.63 | 0.05 | 0.11 | 0.10 | 0.32 | 0.05 | 0.03 | 0.01 | 0.19 | 0.00 | 0.00 | 86.48 |
|  |  |  | BULLIFORM FLABELLATE | 7 | 82.96 | 0.03 | 0.20 | 0.03 | 0.12 | 0.03 | 0.02 | 0.01 | 0.16 | 0.00 | 0.00 | 83.57 |
|  |  |  |  | 24 | 84.60 | 0.03 | 0.13 | 0.05 | 0.14 | 0.04 | 0.03 | 0.01 | 0.17 | 0.00 | 0.00 | 85.20 |
|  |  | Fix-C | Grass silica short cell | 10 | 84.13 | 0.02 | 0.21 | 0.02 | 0.03 | 0.04 | 0.04 | 0.00 | 0.12 | 0.01 | 2.50 | 87.11 |
|  |  |  | ELONGATE | 7 | 83.79 | 0.05 | 0.17 | 0.09 | 0.33 | 0.06 | 0.04 | 0.01 | 0.21 | 0.02 | 2.50 | 87.27 |
|  |  |  | BULLIFORM FLABELLATE | 7 | 82.96 | 0.03 | 0.75 | 0.04 | 0.13 | 0.03 | 0.02 | 0.01 | 0.19 | 0.02 | 2.50 | 86.67 |
|  |  |  |  | 24 | 83.69 | 0.03 | 0.36 | 0.04 | 0.15 | 0.04 | 0.03 | 0.01 | 0.17 | 0.01 | 2.50 | 87.03 |
| *Phyllostachys* | Phyllostachys sulphurea (Carr.) A. et C. Riv | NCA | Grass silica short cell | 9 | 85.40 | 0.02 | 0.73 | 0.05 | 0.08 | 0.09 | 0.03 | 0.03 | 0.14 | 0.00 | 0.00 | 86.58 |
|  |  |  | ELONGATE | 7 | 82.82 | 0.03 | 0.10 | 0.09 | 0.38 | 0.03 | 0.12 | 0.01 | 0.07 | 0.01 | 0.00 | 83.66 |
|  |  |  | BULLIFORM FLABELLATE | 8 | 82.00 | 0.01 | 0.06 | 0.03 | 0.14 | 0.05 | 0.02 | 0.00 | 0.03 | 0.01 | 0.00 | 82.36 |
|  |  |  |  | 24 | 83.51 | 0.02 | 0.33 | 0.06 | 0.19 | 0.06 | 0.05 | 0.01 | 0.09 | 0.01 | 0.00 | 84.32 |
|  |  | Fix-C | Grass silica short cell | 9 | 83.55 | 0.02 | 0.31 | 0.05 | 0.09 | 0.08 | 0.03 | 0.01 | 0.09 | 0.01 | 5.20 | 89.43 |
|  |  |  | ELONGATE | 7 | 83.16 | 0.03 | 0.16 | 0.09 | 0.39 | 0.02 | 0.16 | 0.02 | 0.08 | 0.01 | 5.20 | 89.34 |
|  |  |  | BULLIFORM FLABELLATE | 8 | 85.70 | 0.02 | 0.04 | 0.04 | 0.15 | 0.06 | 0.02 | 0.00 | 0.03 | 0.01 | 5.20 | 91.27 |
|  |  |  |  | 24 | 84.15 | 0.02 | 0.18 | 0.06 | 0.19 | 0.06 | 0.07 | 0.01 | 0.07 | 0.01 | 5.20 | 90.02 |
|  | Phyllostachys nigra (Lodd. ex Lindl.) Munro | NCA | Grass silica short cell | 9 | 82.96 | 0.02 | 0.39 | 0.01 | 0.06 | 0.10 | 0.03 | 0.01 | 0.14 | 0.01 | 0.00 | 83.72 |
|  |  |  |  |  |  |  |  |  |  |  |  |  |  |  |  |  |
|  |  |  | ELONGATE | 8 | 85.48 | 0.03 | 0.07 | 0.01 | 0.23 | 0.04 | 0.06 | 0.01 | 0.14 | 0.01 | 0.00 | 86.08 |
|  |  |  | BULLIFORM FLABELLATE | 7 | 79.99 | 0.03 | 0.05 | 0.02 | 0.14 | 0.03 | 0.01 | 0.01 | 0.14 | 0.01 | 0.00 | 80.44 |
|  |  |  |  | 24 | 83.17 | 0.03 | 0.18 | 0.01 | 0.13 | 0.06 | 0.04 | 0.01 | 0.14 | 0.01 | 0.00 | 83.77 |
|  |  | Fix-C | Grass silica short cell | 9 | 83.87 | 0.03 | 0.17 | 0.02 | 0.08 | 0.10 | 0.02 | 0.01 | 0.13 | 0.01 | 2.20 | 86.63 |
|  |  |  |  |  |  |  |  |  |  |  |  |  |  |  |  |  |
|  |  |  | ELONGATE | 8 | 87.05 | 0.03 | 0.10 | 0.02 | 0.18 | 0.03 | 0.08 | 0.02 | 0.13 | 0.01 | 2.20 | 89.85 |
|  |  |  | BULLIFORM FLABELLATE | 7 | 81.42 | 0.04 | 0.06 | 0.02 | 0.15 | 0.05 | 0.02 | 0.01 | 0.12 | 0.00 | 2.20 | 84.09 |
|  |  |  |  | 24 | 84.22 | 0.03 | 0.11 | 0.02 | 0.13 | 0.06 | 0.04 | 0.01 | 0.13 | 0.01 | 2.20 | 86.96 |
|  | Phyllostachys sulphurea (Carr. ) A. ' Viridis ' | NCA | Grass silica short cell | 10 | 87.71 | 0.03 | 0.12 | 0.04 | 0.03 | 0.01 | 0.03 | 0.02 | 0.18 | 0.01 | 0.00 | 88.19 |
|  |  |  |  |  |  |  |  |  |  |  |  |  |  |  |  |  |
|  |  |  | ELONGATE | 7 | 87.17 | 0.04 | 0.13 | 0.00 | 0.12 | 0.03 | 0.02 | 0.01 | 0.21 | 0.01 | 0.00 | 87.74 |
|  |  |  | BULLIFORM FLABELLATE | 7 | 83.80 | 0.03 | 0.03 | 0.12 | 0.09 | 0.03 | 0.03 | 0.01 | 0.09 | 0.01 | 0.00 | 84.24 |
|  |  |  |  | 24 | 86.41 | 0.03 | 0.09 | 0.05 | 0.07 | 0.02 | 0.03 | 0.01 | 0.16 | 0.01 | 0.00 | 86.91 |
|  |  | Fix-C | Grass silica short cell | 10 | 86.06 | 0.05 | 0.17 | 0.05 | 0.04 | 0.01 | 0.02 | 0.02 | 0.16 | 0.01 | 2.50 | 89.08 |
|  |  |  |  |  |  |  |  |  |  |  |  |  |  |  |  |  |
|  |  |  | ELONGATE | 7 | 84.02 | 0.04 | 0.11 | 0.01 | 0.19 | 0.02 | 0.04 | 0.01 | 0.20 | 0.01 | 2.50 | 87.14 |
|  |  |  | BULLIFORM FLABELLATE | 7 | 83.91 | 0.02 | 0.07 | 0.13 | 0.11 | 0.03 | 0.03 | 0.02 | 0.08 | 0.01 | 2.50 | 86.90 |
|  |  |  |  | 24 | 84.84 | 0.04 | 0.12 | 0.06 | 0.10 | 0.02 | 0.03 | 0.02 | 0.15 | 0.01 | 2.50 | 87.88 |
|  | Phyllostachys heterocycla (Carr.) Mitford cv. Pubescens' | NCA | Grass silica short cell | 10 | 87.82 | 0.02 | 0.04 | 0.01 | 0.04 | 0.07 | 0.04 | 0.01 | 0.12 | 0.01 | 0.00 | 88.18 |
|  |  |  | ELONGATE | 7 | 81.92 | 0.02 | 0.04 | 0.01 | 0.20 | 0.07 | 0.01 | 0.01 | 0.14 | 0.01 | 0.00 | 82.42 |
|  |  |  | BULLIFORM FLABELLATE | 7 | 81.20 | 0.01 | 0.03 | 0.00 | 0.09 | 0.00 | 0.02 | 0.01 | 0.04 | 0.00 | 0.00 | 81.40 |
|  |  |  |  | 24 | 84.17 | 0.02 | 0.04 | 0.01 | 0.10 | 0.05 | 0.03 | 0.10 | 0.01 | 0.01 | 0.00 | 84.52 |
|  |  | Fix-C | Grass silica short cell | 10 | 89.22 | 0.02 | 0.05 | 0.02 | 0.05 | 0.06 | 0.05 | 0.01 | 0.11 | 0.01 | 4.80 | 94.39 |
|  |  |  | ELONGATE | 7 | 79.84 | 0.02 | 0.57 | 0.01 | 0.20 | 0.05 | 0.02 | 0.02 | 0.09 | 0.01 | 4.80 | 85.62 |
|  |  |  | BULLIFORM FLABELLATE | 7 | 89.58 | 0.01 | 0.03 | 0.01 | 0.08 | 0.02 | 0.02 | 0.01 | 0.03 | 0.01 | 4.80 | 94.59 |
|  |  |  |  | 24 | 86.59 | 0.02 | 0.20 | 0.01 | 0.10 | 0.04 | 0.03 | 0.01 | 0.08 | 0.01 | 4.80 | 91.89 |
|  | Phyllostachys praecox C. D. Chu et C. S. Chao 'Prevernalis' | NCA | Grass silica short cell | 10 | 87.51 | 0.03 | 0.17 | 0.04 | 0.06 | 0.02 | 0.03 | 0.01 | 0.13 | 0.01 | 0.00 | 88.01 |
|  |  |  |  |  |  |  |  |  |  |  |  |  |  |  |  |  |
|  |  |  | ELONGATE | 7 | 87.96 | 0.02 | 0.06 | 0.01 | 0.20 | 0.00 | 0.02 | 0.01 | 0.09 | 0.01 | 0.00 | 88.37 |
|  |  |  | BULLIFORM FLABELLATE | 7 | 85.53 | 0.03 | 0.03 | 0.00 | 0.14 | 0.01 | 0.03 | 0.01 | 0.09 | 0.00 | 0.00 | 85.88 |
|  |  |  |  |  |  |  |  |  |  |  |  |  |  |  |  |  |
|  |  |  |  | 24 | 87.06 | 0.03 | 0.09 | 0.02 | 0.13 | 0.01 | 0.03 | 0.01 | 0.10 | 0.01 | 0.00 | 87.49 |
|  |  | NCA | Grass silica short cell | 10 | 87.34 | 0.02 | 0.08 | 0.03 | 0.08 | 0.01 | 0.01 | 0.01 | 0.08 | 0.01 | 5.10 | 92.76 |
|  |  |  |  |  |  |  |  |  |  |  |  |  |  |  |  |  |
|  |  |  | ELONGATE | 7 | 86.82 | 0.02 | 0.05 | 0.00 | 0.17 | 0.01 | 0.03 | 0.01 | 0.10 | 0.01 | 5.10 | 92.32 |
|  |  |  | BULLIFORM FLABELLATE | 7 | 86.00 | 0.03 | 0.03 | 0.00 | 0.16 | 0.02 | 0.01 | 0.01 | 0.08 | 0.00 | 5.10 | 91.43 |
|  |  |  |  |  |  |  |  |  |  |  |  |  |  |  |  |  |
|  |  |  |  | 24 | 86.80 | 0.02 | 0.06 | 0.01 | 0.13 | 0.01 | 0.02 | 0.01 | 0.08 | 0.01 | 5.10 | 92.25 |

**B.** Average elemental compositions and total values (wt.%) (using Fix-C and NCA method) of phytoliths in leaves of bamboo species from three genera.

| Genus | Species | Method | EPMA results | | | | | | | | | | | |
| --- | --- | --- | --- | --- | --- | --- | --- | --- | --- | --- | --- | --- | --- | --- |
|  |  |  | SiO_2_ | Na_2_O | Al_2_O_3_ | MgO | CaO | MnO | Cr_2_O_3_ | P_2_O_5_ | K_2_O | FeO | C | Total |
| *Dendrocalamus* | *Dendrocalamus brandisii* (Munro) Kurz | Fix-C | 85.42 | 0.03 | 0.06 | 0.02 | 0.07 | 0.16 | 0.02 | 0.01 | 0.10 | 0.01 | 2.70 | 88.61 |
|  |  | NCA | 85.42 | 0.02 | 0.05 | 0.02 | 0.06 | 0.16 | 0.02 | 0.01 | 0.10 | 0.01 | 0.00 | 85.87 |
|  | *Dendrocalamus farinosus* (Keng et Keng. f.) Chia et H. L. Fung | Fix-C | 84.25 | 0.06 | 0.46 | 0.02 | 0.10 | 0.02 | 0.02 | 0.01 | 0.12 | 0.01 | 1.40 | 86.46 |
|  |  | NCA | 85.22 | 0.04 | 0.35 | 0.02 | 0.10 | 0.02 | 0.03 | 0.01 | 0.16 | 0.01 | 0.00 | 85.97 |
|  | *Dendrocalamus minor* (McClure) Chia et H. L. Fung | Fix-C | 84.67 | 0.02 | 0.13 | 0.04 | 0.20 | 0.04 | 0.03 | 0.01 | 0.17 | 0.01 | 1.40 | 86.72 |
|  |  | NCA | 83.27 | 0.02 | 0.07 | 0.03 | 0.19 | 0.03 | 0.02 | 0.01 | 0.17 | 0.01 | 0.00 | 83.82 |
|  | *Dendrocalamus giganteus* Munro | Fix-C | 86.75 | 0.05 | 0.04 | 0.03 | 0.13 | 0.04 | 0.03 | 0.01 | 0.20 | 0.01 | 2.40 | 89.69 |
|  |  | NCA | 86.18 | 0.06 | 0.05 | 0.03 | 0.13 | 0.05 | 0.03 | 0.01 | 0.24 | 0.00 | 0.00 | 86.79 |
|  | *Dendrocalamus pachystachys* Hsueh et D. Z. Li | Fix-C | 82.96 | 0.02 | 0.04 | 0.03 | 0.13 | 0.11 | 0.02 | 0.01 | 0.14 | 0.01 | 1.70 | 85.16 |
|  |  | NCA | 83.21 | 0.02 | 0.04 | 0.03 | 0.13 | 0.13 | 0.01 | 0.00 | 0.14 | 0.01 | 0.00 | 83.73 |
|  | *Dendrocalamopsis vario-striata* (W. T. Lin) Keng f. | Fix-C | 84.36 | 0.05 | 0.05 | 0.05 | 0.10 | 0.02 | 0.02 | 0.01 | 0.19 | 0.00 | 2.00 | 86.84 |
|  |  | NCA | 85.42 | 0.04 | 0.05 | 0.04 | 0.10 | 0.02 | 0.03 | 0.01 | 0.21 | 0.01 | 0.00 | 85.92 |
|  | *Dendrocalamus sapidus* Q. H. Dar et D. Y. Huang | Fix-C | 83.82 | 0.04 | 0.08 | 0.02 | 0.09 | 0.05 | 0.03 | 0.01 | 0.12 | 0.01 | 3.30 | 87.57 |
|  |  | NCA | 84.50 | 0.04 | 0.09 | 0.02 | 0.09 | 0.04 | 0.02 | 0.00 | 0.13 | 0.01 | 0.00 | 84.94 |
| *Bambusa* | *Bambusa eutuldoides* McClure | Fix-C | 84.84 | 0.06 | 0.24 | 0.04 | 0.17 | 0.09 | 0.06 | 0.01 | 0.21 | 0.01 | 3.80 | 89.52 |
|  |  | NCA | 85.16 | 0.06 | 0.21 | 0.04 | 0.14 | 0.09 | 0.05 | 0.01 | 0.25 | 0.01 | 0.00 | 86.02 |
|  | *Bambusa chungii* McClure | Fix-C | 86.01 | 0.03 | 0.08 | 0.10 | 0.08 | 0.02 | 0.02 | 0.01 | 0.14 | 0.01 | 2.30 | 88.78 |
|  |  | NCA | 84.78 | 0.04 | 0.13 | 0.09 | 0.08 | 0.02 | 0.02 | 0.01 | 0.18 | 0.01 | 0.00 | 85.35 |
|  | *Bambusa textilis* McClure | Fix-C | 86.05 | 0.02 | 0.30 | 0.02 | 0.11 | 0.07 | 0.03 | 0.01 | 0.11 | 0.01 | 2.50 | 89.24 |
|  |  | NCA | 86.36 | 0.02 | 0.13 | 0.02 | 0.10 | 0.06 | 0.02 | 0.01 | 0.15 | 0.01 | 0.00 | 86.89 |
|  | *Bambusa remotiflora* Kuntze | Fix-C | 85.89 | 0.02 | 0.26 | 0.01 | 0.10 | 0.08 | 0.05 | 0.01 | 0.10 | 0.01 | 1.80 | 88.33 |
|  |  | NCA | 83.74 | 0.03 | 0.39 | 0.01 | 0.10 | 0.08 | 0.08 | 0.01 | 0.13 | 0.01 | 0.00 | 84.57 |
|  | *Bambusa blumeana* J. A. et J. H. Schult. f | Fix-C | 85.53 | 0.06 | 0.04 | 0.01 | 0.12 | 0.01 | 0.03 | 0.02 | 0.20 | 0.01 | 3.01 | 89.04 |
|  |  | NCA | 85.41 | 0.06 | 0.07 | 0.02 | 0.13 | 0.01 | 0.03 | 0.01 | 0.21 | 0.01 | 0.00 | 85.95 |
|  | *Bambusa multiplex* (Lour.) Raeusch. ex Schult. 'Fernleaf' R. A. Young | Fix-C | 85.92 | 0.02 | 0.06 | 0.01 | 0.09 | 0.03 | 0.02 | 0.02 | 0.16 | 0.01 | 3.00 | 89.34 |
|  |  | NCA | 84.80 | 0.02 | 0.12 | 0.01 | 0.08 | 0.03 | 0.03 | 0.01 | 0.18 | 0.01 | 0.00 | 85.28 |
|  | *Bambusa contracta* Chia et H. L. Fung | Fix-C | 83.82 | 0.02 | 0.14 | 0.01 | 0.06 | 0.07 | 0.03 | 0.01 | 0.14 | 0.01 | 1.80 | 86.10 |
|  |  | NCA | 82.62 | 0.02 | 0.14 | 0.01 | 0.06 | 0.05 | 0.04 | 0.01 | 0.14 | 0.01 | 0.00 | 83.11 |
|  | *Bambusa albo-lineata* Chia | Fix-C | 83.69 | 0.03 | 0.36 | 0.04 | 0.15 | 0.04 | 0.03 | 0.01 | 0.17 | 0.01 | 2.50 | 87.03 |
|  |  | NCA | 84.60 | 0.03 | 0.13 | 0.05 | 0.14 | 0.04 | 0.03 | 0.01 | 0.17 | 0.00 | 0.00 | 85.20 |
| *Phyllostachys* | *Phyllostachys sulphurea* (Carr.) A. et C. Riv | Fix-C | 84.15 | 0.02 | 0.18 | 0.06 | 0.19 | 0.06 | 0.07 | 0.01 | 0.07 | 0.01 | 5.20 | 90.02 |
|  |  | NCA | 83.51 | 0.02 | 0.33 | 0.06 | 0.19 | 0.06 | 0.05 | 0.01 | 0.09 | 0.01 | 0.00 | 84.32 |
|  | *Phyllostachys heterocycla* (Carr.) Mitford cv. Pubescens | Fix-C | 86.59 | 0.02 | 0.20 | 0.01 | 0.10 | 0.04 | 0.03 | 0.01 | 0.08 | 0.01 | 4.80 | 91.89 |
|  |  | NCA | 84.17 | 0.02 | 0.04 | 0.01 | 0.10 | 0.05 | 0.03 | 0.01 | 0.10 | 0.01 | 0.00 | 84.52 |
|  | *Phyllostachys nigra* (Lodd. ex Lindl.) Munro | Fix-C | 84.22 | 0.03 | 0.11 | 0.02 | 0.13 | 0.06 | 0.04 | 0.01 | 0.13 | 0.01 | 2.20 | 86.96 |
|  |  | NCA | 83.17 | 0.03 | 0.18 | 0.01 | 0.13 | 0.06 | 0.04 | 0.01 | 0.14 | 0.01 | 0.00 | 83.77 |
|  | *Phyllostachys sulphurea* (Carr. ) A. ' Viridis ' | Fix-C | 84.84 | 0.04 | 0.12 | 0.06 | 0.10 | 0.02 | 0.03 | 0.02 | 0.15 | 0.01 | 2.50 | 87.88 |
|  |  | NCA | 86.41 | 0.03 | 0.09 | 0.05 | 0.07 | 0.02 | 0.03 | 0.01 | 0.16 | 0.01 | 0.00 | 86.91 |
|  | *Phyllostachys praecox* C. D. Chu et C. S. Chao 'Prevernalis' | Fix-C | 86.80 | 0.02 | 0.06 | 0.01 | 0.13 | 0.01 | 0.02 | 0.01 | 0.08 | 0.01 | 5.10 | 92.25 |
|  |  | NCA | 87.06 | 0.03 | 0.09 | 0.02 | 0.13 | 0.01 | 0.03 | 0.01 | 0.10 | 0.01 | 0.00 | 87.49 |

**C.** The elemental content of dominant phytolith morphologies from *Dendrocalamus ronganensi*s leaves determined by EPMA with three methods.

| Method | Phytolith morphology | Carbon values | EPMA results | | | | | | | | | | | |
| --- | --- | --- | --- | --- | --- | --- | --- | --- | --- | --- | --- | --- | --- | --- |
|  |  |  | C | Na_2_O | SiO_2_ | Al_2_O_3_ | MgO | CaO | MnO | Cr_2_O_3_ | P_2_O_5_ | K_2_O | FeO | Total |
| DAC | BLOCKY | 0.00 | 5.97 | 0.02 | 89.25 | 0.01 | 0.05 | 0.06 | 0.00 | 0.00 | 0.00 | 0.03 | 0.02 | 95.41 |
|  |  | 0.00 | 14.25 | 0.00 | 84.33 | 0.02 | 0.08 | 0.16 | 0.00 | 0.00 | 0.00 | 0.07 | 0.00 | 98.91 |
|  |  | 0.00 | 13.19 | 0.03 | 88.83 | 0.02 | 0.01 | 0.18 | 0.03 | 0.02 | 0.00 | 0.10 | 0.00 | 102.40 |
|  |  | 0.00 | 13.86 | 0.01 | 82.77 | 0.01 | 0.04 | 0.18 | 0.02 | 0.07 | 0.00 | 0.01 | 0.00 | 96.96 |
|  |  | 0.00 | 13.19 | 0.02 | 84.37 | 0.03 | 0.03 | 0.20 | 0.02 | 0.00 | 0.00 | 0.10 | 0.00 | 97.96 |
|  |  | 0.00 | 12.60 | 0.00 | 90.70 | 0.00 | 0.04 | 0.14 | 0.00 | 0.00 | 0.03 | 0.04 | 0.00 | 103.55 |
|  | ELONGATE | 0.00 | 12.91 | 0.03 | 91.18 | 0.04 | 0.01 | 0.07 | 0.00 | 0.00 | 0.00 | 0.12 | 0.00 | 104.35 |
|  |  | 0.00 | 22.39 | 0.06 | 64.03 | 0.03 | 0.01 | 0.01 | 0.00 | 0.13 | 0.03 | 0.09 | 0.00 | 86.78 |
|  |  | 0.00 | 12.87 | 0.01 | 83.84 | 0.04 | 0.02 | 0.07 | 0.00 | 0.02 | 0.02 | 0.14 | 0.01 | 97.03 |
|  |  | 0.00 | 53.24 | 0.02 | 84.22 | 0.07 | 0.01 | 0.11 | 0.00 | 0.00 | 0.01 | 0.09 | 0.00 | 137.75 |
|  |  | 0.00 | 12.69 | 0.03 | 87.34 | 0.22 | 0.03 | 0.30 | 0.02 | 0.02 | 0.00 | 0.05 | 0.00 | 100.69 |
|  |  | 0.00 | 12.07 | 0.02 | 90.88 | 0.02 | 0.01 | 0.00 | 0.01 | 0.00 | 0.00 | 0.01 | 0.00 | 103.02 |
|  |  | 0.00 | 11.95 | 0.03 | 73.75 | 0.03 | 0.04 | 0.28 | 0.00 | 0.03 | 0.03 | 0.15 | 0.00 | 86.29 |
|  | Grass silica short cell | 0.00 | 12.53 | 0.01 | 85.77 | 0.24 | 0.19 | 0.22 | 0.03 | 0.02 | 0.02 | 0.10 | 0.02 | 99.13 |
|  |  | 0.00 | 13.78 | 0.01 | 80.76 | 0.05 | 0.07 | 0.09 | 0.00 | 0.02 | 0.01 | 0.04 | 0.01 | 94.82 |
|  |  | 0.00 | 26.17 | 0.01 | 86.54 | 0.05 | 0.18 | 0.09 | 0.03 | 0.01 | 0.00 | 0.06 | 0.02 | 113.16 |
|  |  | 0.00 | 26.47 | 0.04 | 87.94 | 0.05 | 0.05 | 0.19 | 0.08 | 0.04 | 0.00 | 0.13 | 0.00 | 115.00 |
|  |  | 0.00 | 16.17 | 0.00 | 88.82 | 0.06 | 0.12 | 0.12 | 0.02 | 0.04 | 0.00 | 0.08 | 0.00 | 105.42 |
|  |  | 0.00 | 11.37 | 0.01 | 87.39 | 0.04 | 0.01 | 0.04 | 0.00 | 0.03 | 0.02 | 0.34 | 0.00 | 99.26 |
|  |  | 0.00 | 13.67 | 0.02 | 92.05 | 0.06 | 0.09 | 0.09 | 0.08 | 0.01 | 0.00 | 0.12 | 0.01 | 106.20 |
|  |  | 0.00 | 47.30 | 0.01 | 90.92 | 0.05 | 0.02 | 0.02 | 0.00 | 0.00 | 0.03 | 0.14 | 0.02 | 138.50 |
|  |  | 0.00 | 20.36 | 0.02 | 93.70 | 0.03 | 0.07 | 0.15 | 0.03 | 0.04 | 0.00 | 0.06 | 0.01 | 114.46 |
|  |  | 0.00 | 12.87 | 0.01 | 90.46 | 0.03 | 0.05 | 0.06 | 0.00 | 0.00 | 0.00 | 0.01 | 0.00 | 103.50 |
|  |  | 0.00 | 50.09 | 0.02 | 85.23 | 0.17 | 0.06 | 0.15 | 0.06 | 0.04 | 0.00 | 0.02 | 0.01 | 135.86 |
| NCA | BLOCKY | 0.00 | 0.00 | 0.00 | 90.91 | 0.01 | 0.05 | 0.07 | 0.00 | 0.03 | 0.00 | 0.00 | 0.00 | 91.08 |
|  |  | 0.00 | 0.00 | 0.03 | 84.18 | 0.02 | 0.08 | 0.17 | 0.00 | 0.04 | 0.01 | 0.07 | 0.01 | 84.61 |
|  |  | 0.00 | 0.00 | 0.03 | 87.28 | 0.01 | 0.02 | 0.19 | 0.01 | 0.06 | 0.00 | 0.08 | 0.00 | 87.67 |
|  |  | 0.00 | 0.00 | 0.01 | 81.03 | 0.03 | 0.04 | 0.23 | 0.06 | 0.10 | 0.00 | 0.00 | 0.00 | 81.49 |
|  |  | 0.00 | 0.00 | 0.03 | 84.18 | 0.04 | 0.03 | 0.18 | 0.01 | 0.00 | 0.00 | 0.09 | 0.01 | 84.56 |
|  |  | 0.00 | 0.00 | 0.02 | 87.14 | 0.01 | 0.02 | 0.10 | 0.01 | 0.00 | 0.00 | 0.04 | 0.01 | 87.33 |
|  | ELONGATE | 0.00 | 0.00 | 0.03 | 88.10 | 0.02 | 0.02 | 0.09 | 0.00 | 0.01 | 0.00 | 0.14 | 0.00 | 88.40 |
|  |  | 0.00 | 0.00 | 0.08 | 83.19 | 0.12 | 0.02 | 0.04 | 0.02 | 0.03 | 0.00 | 0.14 | 0.00 | 83.64 |
|  |  | 0.00 | 0.00 | 0.10 | 74.23 | 0.06 | 0.00 | 0.05 | 0.04 | 0.08 | 0.02 | 0.14 | 0.00 | 74.72 |
|  |  | 0.00 | 0.00 | 0.01 | 82.86 | 0.33 | 0.02 | 0.14 | 0.00 | 0.01 | 0.00 | 0.05 | 0.01 | 83.43 |
|  |  | 0.00 | 0.00 | 0.08 | 87.57 | 0.15 | 0.01 | 0.17 | 0.01 | 0.41 | 0.00 | 0.16 | 0.02 | 88.57 |
|  |  | 0.00 | 0.00 | 0.03 | 92.75 | 0.05 | 0.00 | 0.00 | 0.03 | 0.00 | 0.04 | 0.04 | 0.01 | 92.96 |
|  |  | 0.00 | 0.00 | 0.02 | 83.94 | 0.00 | 0.13 | 0.20 | 0.00 | 0.02 | 0.00 | 0.09 | 0.00 | 84.40 |
|  | Grass silica short cell | 0.00 | 0.00 | 0.02 | 72.76 | 0.05 | 0.17 | 0.15 | 0.07 | 0.00 | 0.00 | 0.10 | 0.00 | 73.31 |
|  |  | 0.00 | 0.00 | 0.01 | 82.72 | 0.04 | 0.05 | 0.05 | 0.05 | 0.00 | 0.00 | 0.01 | 0.02 | 82.95 |
|  |  | 0.00 | 0.00 | 0.02 | 90.55 | 0.03 | 0.24 | 0.10 | 0.06 | 0.04 | 0.02 | 0.04 | 0.03 | 91.13 |
|  |  | 0.00 | 0.00 | 0.04 | 90.63 | 0.03 | 0.03 | 0.18 | 0.08 | 0.09 | 0.00 | 0.13 | 0.00 | 91.21 |
|  |  | 0.00 | 0.00 | 0.03 | 88.10 | 0.03 | 0.07 | 0.08 | 0.03 | 0.02 | 0.00 | 0.04 | 0.00 | 88.41 |
|  |  | 0.00 | 0.00 | 0.02 | 80.87 | 0.06 | 0.03 | 0.04 | 0.00 | 0.01 | 0.00 | 0.09 | 0.02 | 81.13 |
|  |  | 0.00 | 0.00 | 0.04 | 89.48 | 0.07 | 0.07 | 0.10 | 0.10 | 0.00 | 0.02 | 0.08 | 0.02 | 89.98 |
|  |  | 0.00 | 0.00 | 0.02 | 86.35 | 0.03 | 0.02 | 0.01 | 0.00 | 0.14 | 0.02 | 0.06 | 0.00 | 86.65 |
|  |  | 0.00 | 0.00 | 0.00 | 88.04 | 0.02 | 0.07 | 0.13 | 0.04 | 0.01 | 0.00 | 0.02 | 0.02 | 88.34 |
|  |  | 0.00 | 0.00 | 0.02 | 85.08 | 0.02 | 0.04 | 0.06 | 0.00 | 0.00 | 0.00 | 0.04 | 0.00 | 85.26 |
| Fix-C | BLOCKY | 4.10 | 0.00 | 0.02 | 89.79 | 0.03 | 0.05 | 0.04 | 0.05 | 0.03 | 0.00 | 0.02 | 0.03 | 94.16 |
|  |  | 4.10 | 0.00 | 0.01 | 81.72 | 0.02 | 0.08 | 0.17 | 0.02 | 0.00 | 0.00 | 0.07 | 0.01 | 86.18 |
|  |  | 4.10 | 0.00 | 0.01 | 82.77 | 0.03 | 0.02 | 0.17 | 0.00 | 0.04 | 0.00 | 0.07 | 0.00 | 87.21 |
|  |  | 4.10 | 0.00 | 0.01 | 82.75 | 0.01 | 0.03 | 0.17 | 0.00 | 0.06 | 0.00 | 0.02 | 0.01 | 87.15 |
|  |  | 4.10 | 0.00 | 0.03 | 83.43 | 0.03 | 0.04 | 0.19 | 0.02 | 0.00 | 0.00 | 0.09 | 0.00 | 87.91 |
|  |  | 4.10 | 0.00 | 0.00 | 94.03 | 0.02 | 0.00 | 0.14 | 0.00 | 0.00 | 0.00 | 0.03 | 0.00 | 98.32 |
|  | ELONGATE | 4.10 | 0.00 | 0.03 | 86.88 | 0.03 | 0.02 | 0.06 | 0.01 | 0.00 | 0.00 | 0.20 | 0.00 | 91.33 |
|  |  | 4.10 | 0.00 | 0.04 | 88.09 | 0.05 | 0.00 | 0.06 | 0.07 | 0.00 | 0.02 | 0.08 | 0.02 | 92.54 |
|  |  | 4.10 | 0.00 | 0.02 | 88.81 | 0.10 | 0.02 | 0.04 | 0.00 | 0.06 | 0.03 | 0.05 | 0.00 | 93.23 |
|  |  | 4.10 | 0.00 | 0.02 | 89.35 | 0.08 | 0.06 | 0.21 | 0.02 | 0.01 | 0.00 | 0.11 | 0.18 | 94.13 |
|  |  | 4.10 | 0.00 | 0.07 | 72.43 | 0.15 | 0.01 | 0.24 | 0.01 | 0.17 | 0.00 | 0.15 | 0.00 | 77.33 |
|  |  | 4.10 | 0.00 | 0.03 | 90.70 | 0.02 | 0.01 | 0.04 | 0.00 | 0.00 | 0.01 | 0.03 | 0.00 | 94.95 |
|  |  | 4.10 | 0.00 | 0.04 | 85.72 | 0.03 | 0.02 | 0.20 | 0.00 | 0.00 | 0.01 | 0.12 | 0.01 | 90.24 |
|  | Grass silica short cell | 4.10 | 0.00 | 0.03 | 89.47 | 0.05 | 0.20 | 0.21 | 0.11 | 0.02 | 0.02 | 0.11 | 0.00 | 94.31 |
|  |  | 4.10 | 0.00 | 0.02 | 81.87 | 0.08 | 0.06 | 0.06 | 0.03 | 0.01 | 0.03 | 0.02 | 0.02 | 86.30 |
|  |  | 4.10 | 0.00 | 0.04 | 86.80 | 0.14 | 0.23 | 0.10 | 0.09 | 0.00 | 0.00 | 0.13 | 0.00 | 91.62 |
|  |  | 4.10 | 0.00 | 0.02 | 92.24 | 0.04 | 0.01 | 0.17 | 0.09 | 0.04 | 0.04 | 0.05 | 0.00 | 96.78 |
|  |  | 4.10 | 0.00 | 0.02 | 92.71 | 0.03 | 0.11 | 0.08 | 0.04 | 0.04 | 0.00 | 0.05 | 0.03 | 97.20 |
|  |  | 4.10 | 0.00 | 0.02 | 86.58 | 0.04 | 0.02 | 0.02 | 0.00 | 0.00 | 0.03 | 0.20 | 0.00 | 91.01 |
|  |  | 4.10 | 0.00 | 0.03 | 88.98 | 0.04 | 0.08 | 0.08 | 0.06 | 0.09 | 0.02 | 0.05 | 0.00 | 93.51 |
|  |  | 4.10 | 0.00 | 0.01 | 85.40 | 0.05 | 0.02 | 0.01 | 0.05 | 0.07 | 0.02 | 0.03 | 0.03 | 89.78 |
|  |  | 4.10 | 0.00 | 0.04 | 85.26 | 0.03 | 0.07 | 0.18 | 0.04 | 0.01 | 0.01 | 0.03 | 0.00 | 89.77 |
|  |  | 4.10 | 0.00 | 0.01 | 90.81 | 0.03 | 0.05 | 0.06 | 0.00 | 0.00 | 0.04 | 0.04 | 0.01 | 95.14 |
|  |  | 4.10 | 0.00 | 0.01 | 82.40 | 0.05 | 0.02 | 0.16 | 0.00 | 0.00 | 0.01 | 0.03 | 0.01 | 86.79 |
