# Supplementary material for: Electron probe microanalysis of the elemental composition of phytoliths from woody bamboo species
Source: PLoS One. 2022 Jul 5;17(7):e0270842. doi: 10.1371/journal.pone.0270842 (PMC9255755; doi:10.1371/journal.pone.0270842)
Supplement: Attachment — Submitted filename: Response to Reviewers.docx [file pone.0270842.s002.docx]

**Reponses to reviewers and editors**

Reviewer #1: This paper report an interesting research on the electron probe microanalysis of elemental compositions inside the bamboo phytoliths mounted in the slide. This research is really useful to the plant ecology and paleoenvironmental reconstruction. Before consideration for publication, some questions should be resolved as the followings:

1. L35: what is tree genera?

**Reply:** This was a spelling mistake. “tree genera” should be “three genera”.

2. L76: 10.12%, 3.37%

**Reply:** We have made these changes.

3. L77: PhytOC should have a complete description as first occurrence.

**Reply:** Ok. The complete description of "PhytOC" is "phytolith-occluded carbon".

4. L218, 3.1. The text description is less frequent, and it needs to be strengthened.

**Reply:** We have removed the word “variation” from this section title.

5. L235, 237, Tables 2-3. The chemical composition content lacks units.

**Reply:** We have added this in table captions.

6. L391, 4.3. The author should add some figure and table to show the differences between phytolith morphotypes.

**Reply:** We have provide the Figure 3, table 2 and S1 Table in manuscript, which does exhibit the differences between the morphotypes.

7. L412, 4.4. In present, there is no evident that the elemental compositions of phytoliths have taxonomical significance. Can the authors provide more evidences including the measurements of long saddles, etc.? Otherwise, I suggest the authors delete the relative content.

**Reply:** Thanks for this comment. We have removed the relative content of using the relative abundances of elemental compositions of phytolith to distinguish bamboo species at the genus level, and now only state that the relative abundances of elemental compositions in phytoliths do not show taxonomical significance at the genus level. However, the elemental compositions of phytoliths from these bamboo leaves have ecotypic taxonomical significance in this study. Carnelli et al. investigated the chemical composition of phytoliths from 20 species occurring in subalpine and alpine grasslands, heaths, and woodlands on siliceous bedrock, and found that only woody species produced a high proportion of phytoliths containing aluminum. Thus, we believe the elemental compositions probably do distinguish some plant species, and may have taxonomical significance at the family and/or subfamily level.

8. The format of the reference needs to be corrected.

**Reply:** We have revised them.

9. There are some linguistic errors in the text. It should be polished by an English native speaker.

**Reply:** The manuscript has been polished by an English native speaker.

**Reviewer: 2**

1. Figure 5 the decimal places of total values and SiO_2_ content are different for figure A,B,C. two decimal places are advised.

**Reply:** We have changed the format of these numbers.

2. Line 27-28. “slide mounted” should be “mounted slide”.

Line 35 “tree genera” should be “three genera”

Line 52 “phytolith are normally….”, should be “phytolith morphology is normally”

Line 52. “cells” should be “cell”.

Line 55 “soil and sediments” keep the plural consistent

Line 59 delete the second “to”

Line 73. “lumen phytolith” should be “lumen phytoliths”.

Line 77 change “some research indicates” to “some researches indicate”.

Line 89 “the elemental composition of micro-areas in individual phytolith particles”, “composition” should be in plural.

Line 92 “are” should be changed to “is”

Line 98 “are” should be changed to “is”

Line 108. “phytolith” should be “phytoliths”.

**Reply:** We have made these revisions.

3. Line 112 (iii) What are the characteristic elemental compositions 113 in phytolith is difficult to understand.

**Reply:** We have changed this sentence as “What are the EPMA results of a phytolith particle?”.

4. Line 162. “10 min” should be “10 mins”.

Line 170. “10 min” should be “10 mins”.

Line 203. insert "phytolith particles included" after "20-30".

Line 207 “Dendrocalamus ronganensis” should be in abbreviation, please check through the paper.

Line 219 “phytolith particle were”, change “were” to “was”

Line 231 “phytolith morphologies” should be changed to “phytolith morphotypes”

Line 235 Use phytolith morphotypes

Line 254 “all of Total carbon values”, delete “carbon”, Total should be changed to “total”

Line 282 Please check the use of “CO_2_”

Line 352 “The EPMA of elemental composition in micro-areas of phytolith in mounted slide are …” can be rephrased as “the EPMA results of phytolith in mounted slide are…”

Line 365 to 366, “from these two plant species”, not from two species, use “measured” is Ok

Line 373. “First” should be “Firstly”.

Line 451 change “morphologies” to “morphotypes”

Line 459 change “composition” to “compositions”

Line 464. change " distinguishe" to " to distinguish".

**Reply:** We have made these corrections.

5. Supplemental data

S1 table the caption “the EPMA of phytolith….”should be change to “EPMA results of phytolith”

Table format looks not good.

S2 table the caption “the average elemental compositions”, “the” should be removed.

S3 table the data should be present according to three EPMA methods.

**Reply:** Thanks for your comments. We have made these revisions.
